# Significance of Lewis Phenotyping Using Saliva and Gastric Tissue: Comparison with the Lewis Phenotype Inferred from *Lewis* and *Secretor* Genotypes

**DOI:** 10.1155/2014/573652

**Published:** 2014-03-24

**Authors:** Yun Ji Hong, Sang Mee Hwang, Taek Soo Kim, Eun Young Song, Kyoung Un Park, Junghan Song, Kyou-Sup Han

**Affiliations:** ^1^Department of Laboratory Medicine, Seoul National University College of Medicine, Seoul 110-744, Republic of Korea; ^2^Department of Laboratory Medicine, Seoul National University Bundang Hospital, 173-82 Gumiro, Bundanggu, Seongnam, Gyeonggido 463-707, Republic of Korea

## Abstract

Lewis phenotypes using various types of specimen were compared with the Lewis phenotype predicted from *Lewis* and *Secretor* genotypes. This is the first logical step in explaining the association between the Lewis expression and *Helicobacter pylori*. We performed a study of the followings on 209 patients who underwent routine gastroscopy: erythrocyte and saliva Lewis phenotyping, gastric Lewis phenotyping by the tissue array, and the *Lewis* and *Secretor* genes genotyping. The results of phenotyping were as follows [Le(a−b−), Le(a+b−), Le(a−b+), and Le(a+b+), respectively, in order]: erythrocyte (12.4%, 25.8%, 61.2%, and 0.5%); saliva (2.4%, 27.3%, 70.3%, and 0.0%); gastric mucosa (8.1%, 6.7%, 45.5%, and 39.7%). The frequency of *Le*, *le* ^*59*/*508*^, *le* ^*59*/*1067*^ , and *le* ^*59*^ alleles was 74.6%, 21.3%, 3.1%, and 1.0%, respectively, among 418 alleles. The saliva Lewis phenotype was completely consistent with the Lewis phenotype inferred from *Lewis* and *Secretor* genotypes, but that of gastric mucosa could not be predicted from genotypes. Lewis phenotyping using erythrocytes is only adequate for transfusion needs. Saliva testing for the Lewis phenotype is a more reliable method for determining the peripheral Lewis phenotype of an individual and the gastric Lewis phenotype must be used for the study on the association between *Helicobacter pylori* and the Lewis phenotype.

## 1. Introduction

The Lewis histoblood group system consists of two major antigens, Le^a^ and Le^b^, and three common phenotypes, Le(a−b−), Le(a+b−), and Le(a−b+). The Lewis determinants are oligosaccharides which are synthesized by the sequential addition of sugar units to oligosaccharide chains by fucosyltransferases encoded by* H*,* Secretor*, and* Lewis* genes. The type 2 oligosaccharide chains are expressed mainly on erythrocytes and on vascular endothelial cells, while the type 1 oligosaccharide chains are expressed on the digestive and respiratory tracts and in secretions. The classical Lewis determinants (Le^a^ and Le^b^) are composed of type 1 chains [1]. The Le^a^ antigen is synthesized from a type 1 precursor substrate by* Lewis*-encoded *α*(1,3/1,4)fucosyltransferase, while the Le^b^ antigen is synthesized from a type 1 H substrate by the enzyme.

The eleven* fucosyltransferase* (*FUT*) genes encoding human fucosyltransferases have been isolated [2]. The* FUT1* (*H*) and* FUT2* (*Secretor*) encode *α*(1,2)fucosyltransferases, and the* FUT3*-*FUT9* encode *α*(1,3/1,4 or 1,3)fucosyltransferases.* FUT1*,* FUT2*,* FUT3*, and* FUT6* are polymorphic [1]. *Α*lpha(1,2)fucosyltransferase adds a fucose molecule to the terminal galactose of a precursor to form the H antigen. There are two distinct *α*(1,2)fucosyltransferases in sera and tissues. One is the* H*-encoded fucosyltransferase (H enzyme) and the other is the* Secretor*-encoded fucosyltransferase (secretor enzyme). The H enzyme regulates the expression of the H antigen mainly on erythrocyte membranes and in vascular endothelial cells, while the secretor enzyme regulates the expression of the H antigen mainly on the gastrointestinal epithelial cells and in body fluids such as saliva.

The Lewis antigens are not intrinsic to the erythrocytes but adsorbed onto erythrocyte membranes from plasma. Accordingly, the Lewis phenotyping from erythrocytes is difficult and is sometimes misjudged because of weak hemagglutination due to low titers and low specificities of the reagents. There are two alleles at the* Lewis* locus, the* Le* which encodes a functional fucosyltransferase and the* le* which encodes a nonfunctional enzyme. An individual homozygous for* le* expresses neither Le^a^ nor Le^b^ antigen and has the Le(a−b−) erythrocyte phenotype. Several polymorphisms have been described in the* Lewis* and* Secretor* genes [3–10]. It is conceivable that Lewis antigen expression in digestive organs is biologically much more important than the expression in erythrocytes. The studies on* Helicobacter pylori* suggested that the adherence of* H. pylori* to the human gastric epithelial lining can be mediated by the blood-group antigen-binding adhesion (BabA) that targets human fucosylated blood group antigens type 1 H and Le^b^ [11–13]. The presence of the* babA2* gene, encoding for BabA, in the* H. pylori* genome is crucial for* H. pylori*-related pathogenesis [13].

In this study, various Lewis phenotypes using saliva, erythrocytes, and gastric mucosa were compared with the Lewis phenotype predicted from* Lewis* and* Secretor* genotypes to establish the significance of Lewis phenotyping using saliva and gastric tissue. This is the first logical step in explaining the association between the Lewis expression and* Helicobacter pylori*.

## 2. Materials and Methods

### 2.1. Blood Sample Processing

The subjects were 209 adult patients who underwent routine gastroscopy at a health promotion center because of upper gastrointestinal symptoms. Specimens were collected after the patients had given informed consent. The procedures in this study were in accordance with the National Institutes of Health Bioethics Resources for research on human specimens and the World Medical Association Declaration of Helsinki (Ethical Principles for Medical Research Involving Human Subjects). Peripheral blood was collected in one EDTA tube and one plain tube. The blood of EDTA tube was separated into buffy coat and plasma on the day of blood sampling. The buffy coat was stored at −70°C. DNA for the determination of the* Lewis* and* Secretor* genotypes was extracted from the buffy coat using the Puregene DNA Kit (Gentra, Minneapolis, MN, USA) according to the manufacturer's instructions.

### 2.2. Saliva Sample Processing

Saliva samples were donated for detection of Lewis antigens before undergoing endoscopy. Saliva (5 to 10 mL) was collected in a wide-mouthed test tube. The saliva was centrifuged at 1000 ×g for 10 minutes. The supernatant was transferred to a clean test tube and placed in boiling water bath for 10 minutes to inactivate salivary enzymes. After recentrifuging at 1000 ×g for 10 minutes, the supernatant fluid was diluted with an equal volume of saline and stored at −70°C.

### 2.3. Erythrocyte Phenotyping for the Lewis Antigens

Ortho BioClone 2.0 anti-Le^a^ and anti-Le^b^ monoclonal antibodies (Ortho-Clinical Diagnostics, Inc., Raritan, NJ, USA) were used for hemagglutination tests according to the manufacturer's instructions.

### 2.4. Saliva Testing for the Lewis Phenotypes

The Lewis antigens in saliva were tested by hemagglutination inhibition methods with Lewis antisera. Doubling dilutions of the appropriate blood grouping reagent were prepared beforehand, for the selection of blood grouping reagent dilution. One drop of 3% saline suspension of red cells was added to one drop of each reagent dilution. Le(a+b−) and Le(a−b+) red cells were used to determine Lewis phenotypes. Each tube was centrifuged and examined macroscopically for agglutination. The highest reagent dilution that gives 2+ agglutination was selected. For the hemagglutination inhibition test, one drop of appropriately diluted blood grouping reagent was mixed with one drop of the appropriate saliva and the mixture was incubated for 10 minutes at room temperature. One drop of 3% saline suspension of washed indicator cells was added to each tube. The tube contents were incubated for 60 minutes at room temperature. Each tube was centrifuged and inspected macroscopically for agglutination. Saline control tube was included in each test.

### 2.5. Tissue Array Method

Core tissue biopsies (2 mm in diameter) were taken from individual paraffin-embedded gastric tissues (donor blocks) and arranged in a new recipient paraffin block (tissue array block) using a trephine apparatus (Superbiochips Laboratories, Seoul, Korea). Each tissue array block contained up to sixty cases. Sections of 4 *μ*m were cut from each tissue array block, deparaffinized, and dehydrated.

### 2.6. Gastric Immunostaining for the Lew Phenotypes

Immunohistochemical phenotyping of the gastric tissue specimens for Lewis antigens was performed using a streptavidin peroxidase procedure after an antigen retrieval process using microwaves or autoclaves. The monoclonal antibodies used for detection of gastric Lewis antigens were the antibody to Le^a^ and the antibody to Le^b^ (Signet Laboratories, Inc., Dedham, MA, USA). The same pathologist evaluated all slides blindly. The results of immunostaining were considered to be positive if more than 20% of the cells showed staining.

### 2.7. Genotyping for the* Lewis* Genes

The* Lewis *genotype was determined for the T59G, G508A, and T1067A polymorphic sites. The* le* ^*59/508*^ and* le* ^*59/1067*^ alleles confer very low enzymatic activity relative to the* Le* and* Le* ^*59*^ alleles [4]. The* Le* ^*59*^ allele confers about the same enzymatic activity as the* Le* allele [3].

*(1) PCR-CTPP for the Detection of the T59G Mutation*. The T59G mutation was determined by the polymerase chain reaction with confronting two-pair primers (PCR-CTPP) [14]. The oligonucleotide primers (Bioneer Corporation, Daejeon, Korea) used in the PCR-CTPP were [15] Le59-F1, 5′-CCA TGG ATC CCC TGG GTG-3′; Le59-R1, 5′-CCA CCA GCA GCT GAA ATA GCC-3′; Le59-F2, 5′-CGC TGT CTG GCC GCA CT-3′; Le59-R2, 5′-GAA GGT GGG AGG CGT GAC TTA-3′. PCR was performed in 25 *μ*L reaction mixture containing 10 pmol each of the four primers, 2 *μ*L of DNA, 0.6 units of* Taq* polymerase (Takara, Shiga, Japan), 0.2 mM dNTPs, 2.5 *μ*L 10x PCR buffer, 1.5 mM MgCl_2_, and 2.5 *μ*L of glycerol. PCR conditions were 3 minutes of initial denaturation at 94°C, followed by 35 cycles of denaturing at 94°C for 30 seconds, annealing at 66°C for 30 seconds and extension at 72°C for 30 seconds, and final extension at 72°C for 5 minutes. PCR products underwent electrophoresis in a 3% agarose gel and were stained by ethidium bromide. In the T59G detection by the PCR-CTPP, the 329 bp and 81 bp bands represented the T allele and G allele, respectively. A common band of 373 bp appeared for both alleles.

*(2) PCR-RFLP for the Detection of the G508A and T1067A Mutations*. The G508A and T1067A mutations were determined by the polymerase chain reaction-restriction fragment length polymorphism (PCR-RFLP). For detecting the G508A mutation, genomic DNA was combined with the 508-F (5′-ACT TGG AGC CAC CCC CTA ACT GCC A-3′) and 508-R (5′-TGA GTC CGG CTT CCA GTT GGA CAC C-3′) primers (10 pmol) [6] in 25 *μ*L reaction mixture containing 0.6 units of* Taq* polymerase (Takara, Shiga, Japan), 0.2 mM dNTPs, 2.5 *μ*L 10x PCR buffer, and 1.5 mM MgCl_2_. Thirty cycles (30 seconds at 94°C, 30 seconds at 70°C, and 30 seconds at 72°C) were run, and then the 206 bp products were digested by* Pvu*II enzyme and subjected to separation through 3% agarose gel electrophoresis.

For the T1067A mutation, the first PCR with the primers [4] Le-F (5′-CTC CCG ACA GGA CAC CAC TCC CA-3′) and Le-R (5′-CTC AAG CTT CGT GCC GTG ATG ATC TCT CTG CAC-3′) was carried out in the same PCR buffer as in the PCR for detection of the G508A mutation. Thirty cycles (30 seconds at 94°C, 30 seconds at 70°C, and 45 seconds at 72°C) were run. For the second PCR amplification, the first PCR products were used as the template by 1067-F (5′-CGC TCC TTC AGC TGG GCA CTG GA-3′) and 1067-R (5′-CGG CCT CTC AGG TGA ACC AAG AAG CT-3′) primers [4]. Thirty cycles (30 seconds at 94°C, 30 seconds at 62°C, and 30 seconds at 72°C) were run in the same PCR buffer. The products were digested by* Hin*dIII enzyme and analyzed by 3% agarose gel electrophoresis. The 109 bp product was cleaved into two fragments, 24 and 85 bp, by the digestion.

### 2.8. Genotyping for the* Secretor* Genes

The* Secretor* genotype was determined for the C357T, A385T, and G428A polymorphic sites and the fusion gene. Both* Se* and* Se* ^*357*^ alleles have full enzyme activity. The* se* ^*428*^,* se* ^*385*^,* se* ^*357/385*^, and* se* ^*fus*^ alleles confer little or no enzymatic activity relative to the* Se* and* Se* ^*357*^ alleles [5, 8]. The* se* ^*fus*^ allele is due to fusion of the* Secretor* gene and a pseudogene.

*(1) PCR-CTPP for the Detection of the A385T Mutation and the Fusion Gene*. To detect the A385T mutation and the fusion gene, the genotyping was conducted by means of PCR-CTPP [14]. The primers were as follows [16]: Se5-F0, 5′-TTT CAC TGC CAC CAG CAC CTG-3′; Se385-F1, 5′-ATC AAA GGC ACT GGG ACC CAG-3′; Se385-R1, 5′-GGA CGT ACT CCC CCG GGA T-3′; Se385-F2, 5′-TGG AGG AGG AAT ACC GCC ACT-3′; Se385-R2, 5′-GTC CCC TCG GCG AAC ATG G-3′. Genomic DNA (30–100 ng) was used for each 25 *μ*L reaction mixture containing 0.2 mM dNTPs, 10 pmol each of the five primers, 2.5 *μ*L glycerol, 0.6 units of* Taq* polymerase (Takara, Shiga, Japan), 2.5 *μ*L 10x PCR buffer, and 1.5 mM MgCl_2_. PCR conditions were 3 minutes of initial denaturation at 94°C, followed by 35 cycles of 30 seconds at 94°C, 30 seconds at 61°C, and 30 seconds at 72°C, and final extension at 72°C for 5 minutes. Amplified DNA was visualized on a 2% agarose gel containing ethidium bromide. In the* Secretor* A385T and the fusion genotyping by the PCR-CTPP, the amplified bands with 284 bp, 216 bp, and 353 bp represented the A allele, T allele, and* se* ^*fus*^ allele, respectively. A common band of 460 bp appeared for the A and T alleles.

*(2) PCR-RFLP for the Detection of the C357T and G428A Mutations*. The C357T and G428A mutations were determined by PCR-RFLP. For the detection of the C357T mutation, the first PCR amplification with the primers Se-F (5′-CTC GAA TTC GGG CCT CCA TCT CCC AGC TAA C-3′) and Se-R (5′-CTC AAG CTT GCT TCT CAT GCC CGG GCA CTC-3′) was performed [6]. The Se-F and Se-R primers (10 pmol) were added to 5 *μ*L of genomic DNA in total volume of 50 *μ*L containing 0.2 mM of each dNTP, 0.1 unit of* Taq* polymerase (Takara, Shiga, Japan), 5 *μ*L 10x PCR buffer, and 1 mM MgCl_2_. Thirty cycles (30 seconds at 94°C, 30 seconds at 65°C, and 30 seconds at 72°C) were run. For the second PCR, one *μ*L of the first PCR product was used as the template by the primer sets 357-F (5′-CAG GAT CCC CTG GCA GAA CTA CCA CAT TAA-3′) and 357-R (5′-AGC AGG GGT AGC CGG TGA AGC GGA CGT ACT-3′) [6]. PCR was performed in 25 *μ*L reaction mixture containing 10 pmol each primer, 0.2 units of* Taq* polymerase (Takara, Shiga, Japan), 0.1 mM dNTPs, 2.5 *μ*L 10x PCR buffer, and 4 mM MgCl_2_. The second PCR was carried out under the same conditions as in the first PCR. The 357-F primer created an* Ase*I site in the second PCR product from the mutant allele having C357T, and the 98 bp product was cleaved into two fragments, 28 and 70 bp, by the digestion.

For detection of the G428A nonsense mutation, the first PCR was performed under the same primers and conditions as in the first PCR for detection of the C357T mutation. The second PCR was performed by the primers, 428-F (5′-CGC TTC ACC GGC TAC CCC TGC TTC T-3′) and 428-R (5′-AAC TTC TGG GCC TCC TCC CGC A-3′) [6]. PCR was performed in 25 *μ*L reaction mixture containing 10 pmol each primer, 0.2 units of* Taq* polymerase (Takara, Shiga, Japan), 0.1 mM dNTPs, 2.5 *μ*L 10x PCR buffer, and 4 mM MgCl_2_. Thirty cycles (30 seconds at 94°C, 30 seconds at 60°C, and 30 seconds at 72°C) were run. In case of the product having the G428A mutation, the 107 bp product might be separated into two fragments, 23 and 84 bp, by* Xba*I digestion.

## 3. Results

Lewis phenotypes (from erythrocyte, saliva, and gastric mucosa),* Secretor* genotypes, and* Lewis* genotypes were determined in 209 patients. The number of individuals with erythrocyte phenotype Le(a−b−), Le(a+b−), Le(a−b+), and Le(a+b+) was 26 (12.4%), 54 (25.8%), 128 (61.2%), and 1 (0.5%), respectively, among the 209 individuals. The number of patients with saliva phenotype Le(a−b−), Le(a+b−), Le(a−b+), and Le(a+b+) was 5 (2.4%), 57 (27.3%), 147 (70.3%), and 0 (0.0%), respectively. Lewis antigen expression on gastric mucosa was as follows: Le(a−b−), 17 (8.1%); Le(a+b−), 14 (6.7%); Le(a−b+), 95 (45.5%); Le(a+b+), 83 (39.7%).

The frequency of occurrence of the* Le*,* le* ^*59/508*^,* le* ^*59/1067*^, and* Le* ^*59*^ alleles was 74.6%, 21.3%, 3.1%, and 1.0%, respectively, among 418 alleles examined in total (Table 1). The* le* ^*59/508*^ allele accounted for 87.3% of the* le* alleles, whereas the* le* ^*59/1067*^ allele was 12.7%. The frequency of the* Se*,* Se* ^*357*^,* se* ^*385*^,* se* ^*357/385*^, and* se* ^*fus*^ alleles was 17.7%, 19.4%, 6.7%, 46.7%, and 9.6%, respectively, among 418 alleles examined in total (Table 2).

Tables 1 and 2 summarized whether Lewis phenotype on erythrocytes and known mutations of* Lewis* gene or* Secretor* gene corresponded. In Table 3, various Lewis phenotypes (saliva, erythrocytes, and gastric mucosa) were compared with the Lewis phenotype predicted from* Lewis* and* Secretor* genotypes. The saliva Lewis phenotype was completely consistent with the Lewis phenotype inferred from* Lewis* and* Secretor* genotypes, but the Lewis phenotype in gastric mucosa could not be predicted from* Lewis* and* Secretor* genotypes.

## 4. Conclusion

One out of purposes of the present study was to examine the correspondence between Lewis phenotype on RBCs and known mutations of* Lewis* gene. Moreover various Lewis phenotypes (saliva, erythrocytes, and gastric mucosa) were compared with the Lewis phenotype predicted from* Lewis* and/or* Secretor* genotypes.

Erythrocyte phenotyping through the conventional hemagglutination test has been regarded as a simple way of determining the Lewis antigens. However, in view of our study, erythrocyte phenotyping seems to be incapable of determining accurate Lewis phenotypes. The erythrocyte phenotype is influenced by many factors and may not necessarily reflect someone's* Lewis* and* Secretor* genotypes. The adsorption of glycolipid carrying Lewis activities from plasma onto erythrocytes is sometimes prevented. Some diseases are known to decrease the concentration of circulating Lewis-active glycolipids and cause the incompatible expression of Lewis antigens on erythrocytes. The expression of Lewis antigens has also demonstrated to be affected by the presence of tumors in cancer patients [4].

Lewis phenotyping using erythrocytes is only adequate for transfusion needs. Up to the present, many studies, which have been performed to establish if a disease associates with the Lewis blood group, were not correctly determined the Lewis phenotypes. Therefore, for accurate Lewis phenotyping, alternative methods must be used. In this study, the methods of genotyping and gastric immunohistochemical phenotyping resolved the above problems. However, molecular genotyping only provided an adjunct to phenotyping because the genotyping methods were unable to detect as yet undetermined mutations.

We have calculated Hardy-Weinberg equilibrium (HWE) for our data using Arlequin 3.5.1.3 [17]. The distribution of alleles in the population of our study was deviated from the Hardy-Weinberg equilibrium (*P* < 0.05). Not only our study but also several other studies about* Secretor* and* Lewis* genes shows deviation from HWE [18, 19]. The C357T single nucleotide polymorphism (SNP) is normally present in conjunction with other SNPs of* Secretor* gene. By contrast, the isolated form of the* Se* ^*357*^ allele was present at a relatively high frequency, which indicates the possibility that other combinations of C357T may exist, involving mutations that were not investigated in this population. It is also attributed to the ethnic composition of the sample and the change in population structures. In addition, HWE generally tends to be due to a deficit of heterozygotes for SNP, since the allelic dropout may be the most prevalent genotyping error [20]. Interestingly, our result of HWE calculation revealed deviation due to a deficit of homozygotes. Another possibility of the HWE disequilibrium may be the use of different amplification methods for genotyping, including different DNA polymerase, among studies [14, 16], and it might lead to misjudging the* FUT2* genotype.

When the Lewis phenotype predicted from* Lewis* and* Secretor* genotypes was compared with various Lewis phenotypes (saliva, erythrocytes, and gastric mucosa), the Lewis phenotype obtained from saliva was completely consistent with the Lewis phenotype predicted from* Lewis* and* Secretor* genotypes, but the Lewis phenotype in gastric mucosa was unpredictable. Saliva testing for the Lewis phenotype appears to be a more reliable method for determining the peripheral Lewis phenotype of an individual because the Lewis antigens are not intrinsic to the erythrocytes but adsorbed onto erythrocyte membranes from plasma. Moreover saliva Lewis phenotyping through the hemagglutination inhibition test seems to be able to be used as a simple substituting method for determining the Lewis phenotype by the inference from* Lewis* and* Secretor* genotypes. Because the Lewis expression in gastric mucosa is a different one from the Lewis phenotype by the inference from* Lewis* and* Secretor* genotypes, the gastric Lewis phenotype must be used for the study on the association between the Lewis phenotype and* Helicobacter pylori*.

## Figures and Tables

**Table 1:** *Lewis* genotype and allele frequencies by erythrocyte Lewis phenotype*.

	Le(a−b−) (*n* = 26)	Le(a+b−) (*n* = 54)	Le(a−b+) (*n* = 128)	Le(a+b+) (*n* = 1)	
*Le/Le *	6**	33	69	0	108
*Le/le* ^ 59/508^	14**	20	45	0	79
* Le/le* ^ 59/1067^	2**	1	9	1	13
* Le/Le* ^ 59^	0	0	4	0	4
* le* ^ 59/508^ */le* ^ 59/508^	4	0	1**	0	5
*Le* allele	28	87	196	1	312 (74.6)
*le* ^ 59/508^ allele	22	20	47	0	89 (21.3)
*le* ^ 59/1067^ allele	2	1	9	1	13 (3.1)
*Le* ^ 59 ^allele	0	0	4	0	4 (1.0)

*Values are number or number (percentage).

**Inconsistency between the erythrocyte Lewis phenotypes and the Lewis phenotypes by the inference from *Lewis* genotypes.

**Table 2:** *Secretor* genotype and allele frequencies by erythrocyte Lewis phenotype.

	Le(a−b−) (*n* = 26)	Le(a+b−) (*n* = 54)	Le(a−b+) (*n* = 128)	Le(a+b+) (*n* = 1)	
*Se/Se* ^ 357^	1	0	1	0	2
*Se/se* ^ 385^	1	1*	0	0	2
*Se/se* ^ 357/385^	9	4*	56	0	69
*Se*/*se* ^*fus*^	0	0	1	0	1
*Se* ^ 357^ */Se* ^ 357^	0	1*	1	0	2
*Se* ^ 357^ */se* ^ 357/385^	4	2	34	0	40
*Se* ^357^/*se* ^*fus*^	2	0	33	0	35
*se* ^ 385^ */se* ^ 357/385^	7	18	0	1	26
*se* ^ 357/385^ */se* ^ 357/385^	2	25	1*	0	28
*se* ^357/385^/*se* ^*fus*^	0	3	1*	0	4
*Se* allele	11	5	58	0	74
*Se* ^ 357 ^allele	7	4	70	0	81
*se* ^ 385^ allele	8	19	0	1	28
*se* ^ 357/385 ^allele	24	77	93	1	195
*se* ^*fus*^ allele	2	3	35	0	40

*Inconsistency between the erythrocyte Lewis phenotypes and the Lewis phenotypes by the inference from *Secretor* genotypes.

**Table 3:** Comparison between various Lewis phenotypes and the Lewis phenotype predicted from *Lewis* and *Secretor* genotypes.

	*le/le* and *−/− *			*Le/−* and *se/se *		*Le/−* and *Se/− *				
	*le/le *	*le/le *	*le/le *	*Le/Le *	*Le/le *	*Le/Le *	*Le/Le *	*Le/le *	*Le/le *	
	*Se/Se *	*Se/se *	*se/se *	*se/se *	*se/se *	*Se/Se *	*Se/se *	*Se/Se *	*Se/se *	
	Le(a−b−)			Le(a+b−)		Le(a−b+)				
Lewis phenotype in saliva*										
Le(a−b−)	0	4	1	0	0	0	0	0	0	5
Le(a+b−)	0	0	0	31	26	0	0	0	0	57
Le(a−b+)	0	0	0	0	0	3	78	1	65	147
Le(a+b+)	0	0	0	0	0	0	0	0	0	0
Lewis phenotype on erythrocytes										
Le(a−b−)	0	3	1	3	5	0	3	1	10	26
Le(a+b−)	0	0	0	27	19	1	5	0	2	54
Le(a−b+)	0	1	0	1	1	2	70	0	53	128
Le(a+b+)	0	0	0	0	1	0	0	0	0	1
Lewis phenotype in gastric mucosa**										
Le(a−b−)	0	1	1	0	1	0	3	1	10	17
Le(a+b−)	0	0	0	6	4	0	3	0	1	14
Le(a−b+)	0	3	0	1	1	1	44	0	45	95
Le(a+b+)	0	0	0	24	20	2	28	0	9	83

*The saliva Lewis phenotype through the hemagglutination inhibition test was consistent with the Lewis phenotype inferred from *Lewis* and *Secretor* genotypes.

**The Lewis phenotype in gastric mucosa by immunohistochemistry was not predicted from *Lewis* and *Secretor* genotypes.
